# 25, 50 and 75 years ago

**DOI:** 10.1111/ans.19413

**Published:** 2025-01-24

**Authors:** Julian A Smith

**Affiliations:** ^1^ Department of Surgery Monash University Melbourne Victoria Australia

## 25 years ago

**Vellar ID. Hugh Berchmans Devine: surgical visionary and great Australian ANZ. J. Surg. 2000;70:801–812**.

It is now 75 years since a letter signed by Sir George Syme, Hamilton Russell and Hugh Devine was sent to leading surgeons in Australia and New Zealand inviting them to form an Australasian Association of Surgeons, a body dedicated to improving the standard of surgery as practised in the two countries. This letter proved to be the catalyst that led to the formation of what eventually became the Royal Australasian College of Surgeons, after a previous attempt to form such an Association in 1920 by Professor Louis Barnett had failed. It was Hugh Devine, returning from America in 1925, who played a leading role in the foundation of the College and it is not inappropriate in the year 2000, 75 years after the letter was drafted, to reappraise the life and achievements of this great Australian surgeon.

**McIvor NP, Flint DJ, Gillibrand J, Morton RP. Thyroid surgery and voice‐related outcomes ANZ. J. Surg. 2000;70:179–183**.

Vocal dysfunction in patients with thyroid pathology has been poorly documented, and dysfunction after thyroid surgery is generally reported in terms of recurrent laryngeal nerve or external laryngeal nerve palsy. But voice dysfunction is more complex than simply nerve integrity. The present study reports the incidence of dysphonia in patients presenting for thyroid surgery, and relates postoperative changes in vocal function to recurrent and external laryngeal nerve function, and the surgical handling of the strap muscles. Fifty patients were assessed by Visipitch before and after thyroidectomy. Following surgery, the patients filled out a questionnaire. Overall 26 of 44 patients had no subjective postoperative voice change, while 10 reported subjective deterioration and eight reported subjective improvement in voicing. Postoperative objective assessment of these patients found that 17 were the same, eight refused to come for testing because they felt their voice had not changed, 13 were better and six were worse. Following surgery two patients (4.5%) had temporary recurrent laryngeal nerve palsies (2.5% of nerves at risk), and four patients (10%) suffered external laryngeal nerve palsies. Division of strap muscles was not detrimental to voicing. Six patients were lost to follow‐up. Fifteen patients (34%) presented with vocal abnormalities, six (40%) of whom improved postoperatively. Patients may have voicing abnormalities before thyroid surgery is performed. Surgery may improve or worsen the voice irrespective of the pre‐operative voice status.

## 50 years ago

**Hunter R, Oakley JR. Hot nodules of the thyroid gland ANZ. J. Surg. 1975;45:191–196**.

The Department of Nuclear Medicine, Institute of Medical and Veterinary Science, Royal Adelaide Hospital, first made thyroid scans available in May 1968. This paper reviews the hot nodules found in these scans during the period March 1969 to November 1972. The significance of the single hot nodule is discussed, and an attempt is made to assess the accuracy of tests used to diagnose the autonomous hot nodule. In conclusion:

1. The thyroid scan, alone or in combination with the clinical findings, is not reliable in differentiating between a hyper‐functioning thyroid adenoma and a hyper‐functioning nodule within a multinodular goitre.
2. The TSH stimulation test and the T_3_ suppression test, although of value in proving the autonomy of hot nodules, do not help in differentiating between a benign adenoma and **a** nodule in a multinodular goitre.
3. It is possible for an area within a multinodular goitre to function autonomously, or alternatively the TSH stimulation and T_3_ suppression tests as presently performed are unsatisfactory.

**Barraclough BH, Reeve TS. Postoperative complications of thyroidectomy ANZ. J. Surg. 1975;45:21–29**.

A comparison is presented of the complications found in two series each consisting of 331 consecutive patients undergoing thyroidectomy In the same Unit 10 years apart. The overall incidence of postoperative complications has been reduced, particularly in the group of patients having thyroidectomy for thyrotoxicosis, following which it is now no greater than after thyroidectomy for benign non‐toxic goitre. The techniques used to try to reduce the postoperative complication rate are discussed. The incidence of permanent recurrent laryngeal nerve palsy has been reduced to 0.3% and of permanent hypoparathyroidism to 1.2%. The overall incidence of complications causing permanent disability is now 3.9%. Further efforts must be made to reduce the incidence of hypoparathyroidism, particularly in those patients having total thyroidectomy for malignancy. Improved preoperative assessment and treatment have helped to reduce the complication rate in thyrotoxic patients to the same level as occurs with other benign thyroid disease. Postoperative complications of any type can be minimized if at the time of operation, the patient has been appropriately prepared and assessed to the satisfaction of the surgeon and his physician colleagues, and if any necessary resuscitation and postoperative nursing care can he performed in ideal conditions.

## 75 years ago

**Poate HRG. Malignant disease of the thyroid ANZ. J. Surg. 1950;20:114–125**.

In summary:

1. Carcinoma of the thyroid is much more common than is generally thought.
2. Age is no criterion as to its possible development.
3. It is most commonly found in association with or super‐imposed upon a single adenoma in the thyroid. The incidence in males is nearly double that in females in these cases.
4. It is seldom met in association with acute thyrotoxicosis but some cases of carcinoma of the thyroid may present a low‐grade chronic thyrotoxicosis.
5. It does occur occasionally in multinodular goitres and is usually evidenced by a rapid increase in growth with hardness in one area.
6. There are no reliable positive signs or symptoms to aid early diagnosis.
7. The best prophylaxis is early removal of all single adenomata irrespective of age and of the multiple adenomatous gland in which recent change has developed.
8. Excision of the affected area, lobectomy or even total thyroidectomy are the correct surgical procedures. Enucleation is to be avoided. Complete examination of the whole gland is advisable at operation.
9. In late stage cases without metastases resection of the tumour should be attempted and followed by deep X‐ray therapy.
10. It is seldom necessary to carry out a block dissection of the cervical glands.
11. In the papilliferous carcinomata, all involved glands should be removed along with a lobectomy on the involved side.
12. All cases except the papilliferous tumours should be given adequate deep X‐ray therapy. Even in late stage cases, expectancy of life is increased and occasionally prolonged remission is met with.
13. The injudicious use of iodine in cases of adenomatous goitre may very possibly play a part in stimulating the onset of carcinoma.
14. It is most unlikely that ‘thio’ compounds used for the control or cure of thyrotoxicosis will lead to the increase in carcinoma of the thyroid but all persistently enlarged glands after its use and all cases of toxic nodular goitre should be advised to undergo operation.

